# Genkwanin Prevents Lipopolysaccharide-Induced Inflammatory Bone Destruction and Ovariectomy-Induced Bone Loss

**DOI:** 10.3389/fnut.2022.921037

**Published:** 2022-06-23

**Authors:** Xin Fu, Xiaochen Sun, Chenxi Zhang, Nanning Lv, Huan Guo, Chunlei Xing, Juan Lv, Jiwen Wu, Xiaoli Zhu, Mingming Liu, Li Su

**Affiliations:** ^1^Institute of Translational Medicine, Shanghai University, Shanghai, China; ^2^Center for Molecular Recognition and Biosensing, School of Life Sciences, Shanghai University, Shanghai, China; ^3^School of Medicine, Shanghai University, Shanghai, China; ^4^Lianyungang Second People’s Hospital, Lianyungang, China; ^5^Lianyungang Clinical School of Xuzhou Medical University, Lianyungang, China

**Keywords:** genkwanin, osteoclast, postmenopausal osteoporosis, MAPK, ovariectomy

## Abstract

**Objectives:**

The first objective of this study was to probe the effects of genkwanin (GKA) on osteoclast. The second goal of this study was to study whether GKA can protect lipopolysaccharide (LPS) and ovariectomized (OVX) induced bone loss.

**Materials and Methods:**

Various concentrations of GKA (1 and 10 mg/kg) were injected into mice. Different concentrations of GKA (1 and 5 μM) were used to detect the effects of GKA on osteoclast and osteoblast.

**Key Findings:**

GKA attenuated the osteoclast differentiation promoted by RANKL and expression of marker genes containing *c-fos, ctsk* as well as bone resorption related gene *Trap* and to the suppression of MAPK signaling pathway. In addition, GKA induced BMMs cell apoptosis *in vitro*. Moreover, GKA prevented LPS-induced and ovariectomized-induced bone loss in mice.

**Conclusion:**

Our research revealed that GKA had a potential to be an effective therapeutic agent for osteoclast-mediated osteoporosis.

## Introduction

Bones are living tissue in a dynamic balance, which is continuously remodeled to adapt its structure even in adults (1, 2). The remodeling process depends on the coordination and interaction between osteoclast and osteoblast (3). The pathological change of the bony skeleton results in a severe imbalance between bone-formation and bone-resorption (4). Osteoporosis is caused by excessive bone resorption, which was involved in activation of osteoclasts (5–8). Therefore, the treatment for osteoporosis primarily focuses on inhibiting the proliferation and function of osteoclasts (9). To date, several anti-osteoporotic drugs, such as bisphosphonates and denosumab, have been developed and used in clinic, which have shown positive effects. However, severe adverse reactions including osteonecrosis of the jaw and increased risk of breast cancer have compromised the health of patients, limiting the use of them in clinical practice (10, 11). However, severe adverse reactions including osteonecrosis of the jaw and increased risk of breast cancer have compromised the health of patients, limiting the use of bisphosphonates and denosumab in clinical practice (12, 13). In addition, there are some anabolic drugs, such as parathyroid hormone analogues (PTH) approved by Food and Drug Administration (FDA), which can accelerate bone formation well (14). Although PTH significantly promotes bone formation, it activates osteoclasts and further accelerates bone resorption (15). Once the process of bone resorption surpasses that of bone formation, excessive bone resorption will occur (16).

Estrogen plays an important role in bone formation. Under physiological conditions, estrogen not only promotes the differentiation of osteoblasts and the apoptosis of osteoclasts, but also inhibits the secretion of inflammatory factors by immune cells such as T lymphocytes (17). When estrogen is deficient, the increase of inflammatory factors leads to the activation of osteoclasts and bone resorption, resulting in bone loss (18, 19). In mouse bone marrow-derived macrophages and RAW264.7 cells, estrogen can inhibit LPS-induced TNF release through suppressed NF-κB pathway and NO release (20, 21).

Under stimulation of some cytokines, osteoclasts are derived from monocytes or macrophages. Especially, RANKL is involved in the formation and fusion of osteoclasts (22–24) and M-CSF has an essential influence on cell multiplication and survival (25). The several intracellular signaling pathways involved in NF-κB, MAPK, and AP-1 (26, 27) are activated by the stimulation of RANKL and M-CSF. The expression of activated T-cell cytoplasmic 1 (NFATc1) was upregulated, which induces a range of process such as osteoclast formation, fusion, and bone resorption (25, 28). Thus, the transduction of intracellular signaling pathway may become a new strategy to prevent osteoporosis.

Recently, genkwanin (GKA), as the representative bioactive non-glycosylated flavonoid (29, 30), has been proved to protect adjuvant-induced arthritis (AIA) rat model from inflammation and joint destruction through suppressing JAK/STAT as well as NF-κB signaling pathways (31). Moreover, it is indicated that GKA can regulate MiR-101/MKP-1/MAPK pathway to generate anti-inflammatory effects in LPS-induced macrophages (32). Additionally, GKA can enhance host immunity and decrease the inflammatory cytokine levels to prevent tumor. To sum up, immunomodulatory, anti-bacterial, anti-plasmodial, radical scavenging, and chemopreventive activities of GKA have been proved (33–35). However, less research has been investigated to focus on the effect of GKA on bone homeostasis. In this research, we probed the act and mechanism of GKA in ovariectomize-induced mice. The results suggested that GKA can not only suppress RANKL-induced osteoclast differentiation *in vitro*, but also protect bone loss from OVX-induced *in vivo*. Moreover, this protective effect of GKA was owing to the inhibition of the MAPK pathway. Hence, GKA has a potential to be a drug candidate against postmenopausal osteoporosis.

## Materials and Methods

### Agents

Genkwanin (purity ≥98% by HPLC) was supplied by Chengdu DeSiTe Biological Technology Co., Ltd (Chengdu, China). Dimethyl sulfoxide (DMSO) was furnished by Beyotime Biotechnology (Jiangsu, China). Hyclone (GE Healthcare, Chicago, IL, United States) provided the alpha modification of minimal essential liquid medium (α-MEM). Moreover, fetal bovine serum, L-glutamine, as well as penicillin and streptomycin (P/S) were supplied by Gibco (Thermo Fisher Scientific, Waltham, MA, United States). Recombinant mouse M-CSF and RANKL were obtained from R&D Systems (Minneapolis, MN, United States). Tartrate-resistant acid phosphatase (TRAP) staining kit was provided by Joytech Bio Inc (Zhejiang, China). Specific primary antibodies against p-p38 (Thr180/Tyr182; #4511), p38 (#8690), p-IκBα (2859), IκBα (#4812), p-IκBα (2859), p-SAPK/JNK (Thr183/Tyr185; #4668), SAPK/JNK (#9252), p-ERK1/2 (Thr202/Tyr204; #4370), ERK1/2 (#4695), and GAPDH (#5174) were purchased from Cell Signaling Technology (Danvers, MA, United States). Abcam (Cambridge, United Kingdom) provided the antibody against c-Fos (ab190289), goat anti-rabbit IgG antibody (IRDye 800CW; ab216773) as well as Phalloidin-iFluor 488 reagent (ab176753). Furthermore, the Santa Cruz Biotechnology (Dallas, TX, United States) offered NFATc1 antibody (7A6; sc-7294). Besides, Takara Bio Inc. (Shiga Prefecture, Japan) supplied the PrimeScript RT Master Mix (#RR036A) as well as TB Green Premix Ex Taq (RR420A). Mouse TNF-α and IL-6 ELISA kits were acquired from Biolegend Co. (San Diego, California, United States). Lipopolysaccharide (LPS) was purchased from Sigma-Aldrich (#L2880). Excell Technology Co. (Shanghai, China) supplied the mouse IL-1β ELISA kit. IL-1β (HY-P7073) was purchased from MedChemExpress (NJ, United States). Alkaline Phosphatase Color Development Kit and 5-bromo-4-chloro-3-indolyl phosphate (BCIP)/nitro blue tetrazolium (NBT) Alkaline Phosphatase Color Development Kit (C3206) were derived from Beyotime Biotechnology (Shanghai, China).

### Cell Isolation and Culture

Fresh bone marrow macrophages (BMMs) and bone marrow stroma cells (BMSCs) were separated carefully from lower limbs of 6-week-old C57 mice using the reported method (36). Then, cells were incubated in α-MEM, which added 1% P/S and 10% FBS. BMMs were cultivated in α-MEM adding 25 ng/ml M-CSF and replaced the medium every 3 days.

### Cell Viability Assay

To evaluate the influence of GKA on the viability of BMSCs and BMMs, survival rate in presence of various concentrations of GKA was measured by CCK-8 (Dojindo, Kumamoto, Japan). First, approximately 8 × 10^3^ BMMs were seeded into 96-well plates with 100 μl complete α-MEM (containing 10% FBS, 1% P/S, and 25 ng/ml M-CSF) for 1 day, and the medium was substituted by fresh medium. BMSCs were seeded into 96-well plates at a density of 6 × 10^3^ cells/well and allowed to adhere overnight. Subsequently, after incubating for 48, 72, and 96 h, respectively, with different concentrations of GKA (0, 0.625, 1.25, 2.5, 5, 10, 20, 40, and 80 μM), 10 μl CCK-8 reagent was added. Finally, the absorbance of 450 nm was detected after incubating for 4 h.

### Detection of Apoptosis in Bone Marrow Macrophages

Approximately 1.5 × 10^5^ BMMs were seeded in 6-well plate and cultured 12 h for cell adhesion. The following day, cells were treated to various concentrations of 0, 10, 20, and 40 μM of GKA for 48 h. After that, BMM cells were harvested and washed with fresh PBS for 3 times. Then, the apoptotic reagents (300 μl binding buffer, 2 μl PI, and 2 μl Annexin V-FITC for each tube) were used for incubating cells for 10 min. Finally, cells were washed three times and the quantity of apoptotic cells was determined by flow cytometry.

### Osteoclastogenesis and Tartrate-Resistant Acid Phosphatase Staining

The BMMs were seeded into 96-well plates at a density of 8 × 10^3^ with complete α-MEM, which contained 1% P/S, 10% FBS, 25 ng/ml M-CSF, and 50 ng/ml RANKL with different concentrations of GKA for 1 week. The medium was replaced every 48 h. To assess osteoclasts quantity, these cells were stained using tartrate-resistant acid phosphatase (TRAP) after fixation with 4% paraformaldehyde (PFA) for 15 min. Finally, the cells were observed with a light microscope. TRAP^+^ cells with >3 nuclei were quantified using the ImageJ software. The time-dependent effect of GKA on osteoclast formation was investigated using GKA at 5 μM. The cells were divided into the following groups: D5–D6: GKA was added on day 5 for 48 h; D4–D6: GKA was added on day 4 for 72 h; D3–D5: GKA was added on day 3 for 48 h; D1–D3: GKA was added on day 1 for 48 h; D1–D6: GKA was added on the first day and removed on the sixth day. Finally, cells with >3 nuclei were imaged and analyzed using a method described previously.

### Fibrous Actin Ring Immunofluorescence

The BMMs were seeded and cultured into 96-well plates with complete α-MEM, which added 25 ng/ml M-CSF, 50 ng/ml RANKL; in the meantime, various concentrations of GKA were added to treat cells for 5 days or until mature multinucleated osteoclasts are formed. Finally, 4% PFA was supplied to fix these cells for 15 min and stained with rhodamine-conjugated phalloidin (Cytoskeleton, Denver, CO, United States) for detecting fibrous actin (F-actin) ring and with DAPI for detecting nuclei. The cells were visualized using fluorescence microscope and quantified using the ImageJ software.

### Resorption Pit Assay

To evaluate function of osteoclasts, the hydroxyapatite resorption assay was conducted (37, 38). Six-well plates were used to seed and culture BMMs at a density of 8 × 10^4^ cells/well in the medium as before for 3 days. The cells were trypsinized and mixed with various concentration of GKA, and then the cells were seeded in hydroxyapatite-coated 96-well plates at a density of 8 × 10^3^. The cells were removed by 5% sodium hypochlorite solution after 4 days. The resorption pit was detected using optical microscope. Resorption areas were measured using the ImageJ software.

### Alkaline Phosphatase Staining

Bone marrow stroma cells were isolated carefully from lower limbs of 6-week-old mice and immediately cultured in complete α-MEM (15% FBS and 1% P/S). After that, 1 × 10^6^ cells per well were seeded into a 12-well plate with or without GKA using osteogenic medium, with excessive addition of 100 mM L-ascorbic acids, 10 mM b-glycerophosphate, and 100 nM DMEM for 1 week. The medium was replaced by half of the osteogenic medium every second day. On day 7, the cells were fixed with 4% PFA for 10 min before ALP staining (refer to protocol).

### RNA Preparation and Real-Time PCR

Bone marrow macrophages were cultured for 1 week with 50 ng/ml RANKL and various concentrations of GKA, and then total RNA was extracted from cells using Trizol solution (Ambion, Thermo Fisher Scientific) on the basis of the instructions. When RNA was reverse-transcripted into cDNA, 1 μg RNA was used and 4 μl 5× PrimeScript RT Master Mix and RNase-free ddH_2_O were added to make up the total volume to 20 μl. The primers supplied for qPCR are given in Table 1. The reaction mixture for qPCR of each sample was composed of 5 μl cDNA, 10 μl TB Green Premix Ex Taq, 0.6 μl of each primer (10 μM), and 3.8 μl RNase free ddH_2_O. The PCR program comprises 95°C (5 min) initially, followed by 40 cycles of 95°C (10 s), 60°C (20 s), and 72°C (30 s). The 2^–ΔΔ^ ^CT^ method was utilized to quantify the expression of relative target gene.

**TABLE 1:** Primer sequences used for RT-PCR.

Genes	Sequence (5′–3′)	
*Trap*	F: TCCTGGCTCAAAAAGCAGTT	R: ACATAGCCCACACCGTTCTC
*c-fos*	F: CCAGTCAAGAGCATCAGCAA	R: AAGTAGTGCAGCCCGGAGTA
*ctsk*	F: CTTCCAATACGTGCAGCAGA	R: TCTTCAGGGCTTTCTCGTTC
*Atp6v0d2*	F: AAGCCTTTGTTTGACGCTGT	R: TTCGATGCCTCTGTGAGATG
*DC-STAMP*	F: AAAACCCTTGGGCTGTTCTT	R: AATCATGGACGACTCCTTGG
*Tnf*α	F: AGTGACAAGCCTGTAGCCC	R: GAGGTTGACTTTCTCCTGGTAT
*Il-6*	F: TGTATGAACAACGATGATGCACTT	R: ACTCTGGCTTTGTCTTTCTTGTTATCT
*GAPDH*	F: ACCCAGAAGACTGTGGATGG	R: CACATTGGGGGTAGGAACAC
*OPG*	F: CCGAGGACCACAATGAACAAGT	R: CTGGGTTGTCCATTCAATGATG
*Rankl*	F: CTGGGCCAAGATCTCTAACATGA	R: GGTACGCTTCCCGATGTTTC

### Western Blot

To investigate how GKA affects the pathway of osteoclasts formation, first, BMMs were starved for 2 h, and then 5 μM GKA was added for 2 h treatment. Furthermore, 50 ng/ml RANKL were supplied to stimulate cells for different time points (5, 10, 20, 30, and 60 min). To estimate the effect of GKA on c-Fos and NFATc1, the BMMs were treated in RANKL-containing medium with GKA for 1, 3, and 5 days. RIPA lysis buffer containing PMSF was applied to extract proteins on ice, and then 25 μg protein lysates were added into the 12% SDS-PAGE gels to separate various proteins. Furthermore, the procedure was performed as shown previously (39). Relative protein expression was analyzed through calculating the gray-scale blots using the ImageJ software.

### Ovariectomized-Mice Model

A total of 24 female 12-week-old C57BL/6 mice were randomly divided into 4 groups (*n* = 6) as follows: sham (mock surgery with PBS injection), OVX (with PBS injection), low (OVX + GKA 1 mg/kg), and high (OVX + GKA 10 mg/kg). Animals in all the groups except sham group were ovariectomized bilaterally. One week after surgery, the low and high groups were treated with GKA. The GKA treatment is followed for every 2 days for 4 weeks. At the same time, OVX and sham groups were treated with PBS. Finally, euthanasia was applied to sacrifice mice and their tibias were harvested to fix in 4% PFA for subsequent detection and analysis.

### Lipopolysaccharide-Induced Calvarial Osteolysis Model

Eighteen 8-week-old male C57BL/6 mice were randomly assigned into 3 groups (*n* = 6 mice per group): sham control (mock operation with PBS injection), LPS only (5 mg/kg body weight), LPS with GKA (10 mg/kg body weight). Mice were anesthetized with the use of isoflurane gas, and then, all mice received subcutaneous injection over the sagittal midline suture of the calvarium. On day 0, sham control and LPS group received PBS or LPS and PBS injection, respectively, whereas GKA treatment groups received LPS and GKA injections together. PBS and GKA were injected every other day over a 7-day period after which all mice were sacrificed. Calvarial bones were harvested, fixed in 4% PFA, and processed for micro-CT and histological assessment.

### Micro-Computed Tomography Analysis

The high-resolution micro-computed tomography (μCT) was used to scan each left tibia and isometric resolution was set at 9 μm. The x-ray energy was set to 80 kV and 100 μA. The three-dimensional (3D) imaging was recurred using the SkyScan NRecon procedure and SkyScan CTAn software (Bruker, Billerica, MA, United States). Trabecular bone analysis was performed in the specific zone of 0.5 mm below the growth plate of the tibia with a height of 1 mm. Trabecular morphometry was evaluated by calculating the bone volume/tissue volume (BV/TV), bone mineral density (BMD), number of trabeculae (Tb.N., mm^–1^), trabecular separation (Tb.Sp., mm), cortical thickness (Ct.Th), and cortical bone mineral content (Ct.BMC).

### Histological Examination

Tibia tissues were first fixed in 4% formaldehyde for 48 h, and then decalcified with 10% ethylenediaminetetraacetic acid (EDTA) for half a month. Further procedures were performed as described according to the protocol. Briefly, the samples were embedded in paraffin and sectioned into slides to 5 mm thickness. Subsequently, hematoxylin and eosin (H&E) staining and TRAP staining were carried out and then were observed through a high-quality microscope. Quantitative histomorphometric analysis was performed using the OsteoMeasure software version 3.2.1. Moreover, to probe the effect of GKA on the formation of osteoblasts, samples were stained with von Kossa to detect the calcium nodule. Calcein and Alizarin Red S were injected at 10 mg/kg according to the body weight on 10th and 3rd days before the mice were sacrificed, respectively. The tibias were collected, fixed, and cut into 5 μm sections. The samples were observed using a laser confocal microscope. Trabecular bone analysis was performed in a specific region of 0.5 mm below the growth plate of the tibia with a height of 1 mm. Histomorphometry analysis was evaluated by measuring BV/TV, trabecular thickness (Tb.Th., mm), number of trabeculae (Tb.N., mm^–1^), and trabecular separation (Tb.Sp., mm). Mineral apposition rate (MAR) was measured by dividing the distance between two labels by the time between dye injections (40).

### Detection of Cytokines in Mice Serum

The mouse blood was centrifuged with 5,000 *g* for 15 min at 4°C, and the upper layer liquid was collected and then stored at −80°C. The levels of IL-6, TNF-α, and IL-1β in the serum was measured by ELISA detection kit and calculated according to an appropriate standard curve with a picogram per milliliter of serum.

### Statistical Analysis

Three independent experiments were used to obtain the representative results. The difference between various groups was assessed with GraphPad Prism 5.0 (San Diego, CA, United States) using Student’s *t*-test or one-way analysis of variance (ANOVA). *P*-value < 0.5 was considered statistically significant.

## Results

### Genkwanin-Induced Bone Marrow Macrophages Apoptosis and Suppressed RANKL-Induced Osteoclastogenesis *in vitro*

**FIGURE 1:**
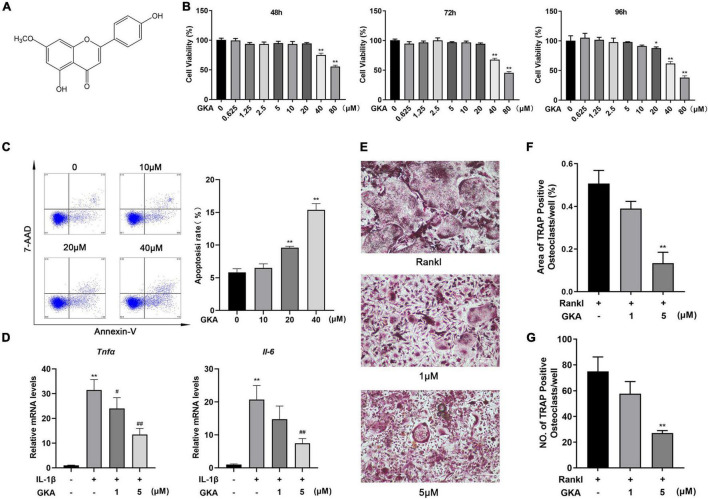
Genkwanin (GKA) induced cell apoptosis and prevented osteoclastogenesis stimulated by RANKL *in vitro*. **(A)** Structural formula of GKA. **(B)** Cell viability was investigated by CCK-8 assay at different time points. **(C)** BMMs were treated with different concentration of GKA for 48 h, and the cell apoptosis rate was examined by flow cytometry. **(D)** BMMs were induced by IL-1β (10 ng/ml) and treated with GKA for 6 h, and *Tnf*α and *Il-6* were detected by RT-PCR. **(E)** BMMs were treated with various concentrations of GKA. Representative image of TRAP-positive osteoclasts (scale bar, 500 μm). **(F,G)** The number and area of TRAP-positive multinucleated osteoclasts (>3 nuclei) were calculated. The data were described as mean ± SD (*P < 0.05, **P < 0.01, relative to the control group. ^#^*P* < 0.05, ^##^*P* < 0.01, GKA treatment group relative to the positive group, analyzed by one-way ANOVA), *n* = 3. Error bars refer to standard deviations from three replicates.

The effects of GKA on the proliferation of osteoclast precursor cells were evaluated through CCK-8 detection (Figure 1A). Treatment of 20 μM GKA for 48 or 72 h had a minimal effect on the cell viability of BMMs. However, the cell viability was decreased after treatment with 40 and 80 μM GKA for 48, 72, and 96 h (Figure 1B). The results suggested that the viability of BMMs was not affected by 20 μM GKA. In addition, the cell apoptosis and RANKL-induced osteoclastogenesis by GKA were investigated. The results showed that 40 μM GKA-induced BMMs apoptosis after being cultured for 48 h (Figure 1C). While the total number of TRAP^+^ osteoclasts was decreased with the treatment of GKA in a dose-dependent manner (Figure 1E). Especially, TRAP^+^ osteoclasts decreased from 75 ± 5 to 27 ± 2 after being treated with 5 μM GKA (Figures 1E,G). Moreover, TRAP staining images showed that the area of TRAP^+^ osteoclasts was significantly reduced with the treatment of GKA (Figure 1F). These suggested that GKA-induced BMM cell apoptosis and suppressed RANKL-induced osteoclastogenesis.

### Genkwanin Suppressed RANKL-Induced Osteoclast Differentiation *in vitro*

**FIGURE 2:**
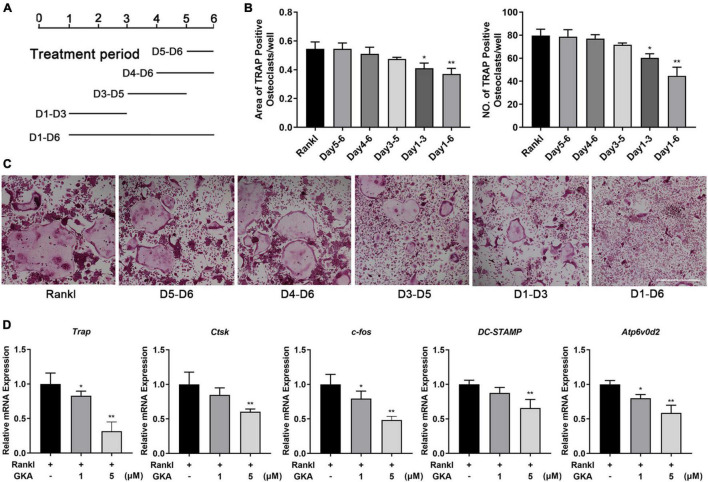
GKA decreased osteoclast differentiation during the early stages of osteoclast differentiation and gene expression *in vitro*. **(A)** Schematic diagram of the GKA treatment time. **(B)** The quantity and area of osteoclasts were determined. **(C)** BMMs was treated with 5 μM GKA for the indicated period of time. Representative image of TRAP-positive osteoclasts (scale bar, 500 μm). **(D)** The relative expression of osteoclast marker genes (*Trap, ctsk, c-fos, Atp6v0d2*, and *DC-STAMP*) following GKA treatment was quantified by RT-PCR (*n* = 3). The data were described as mean ± SD (*P < 0.05, **P < 0.01, relative to the control group, analyzed by one-way ANOVA).

To investigate which phase of osteoclast differentiation was predominantly inhibited by GKA, the BMMs were cultured with indicated concentration of GKA at five different time points (Figure 2A). The results in Figure 2C revealed that GKA has a significant inhibitory effect on osteoclast formation in the early stage. Moreover, compared with the control group, both areas and numbers of TRAP-positive osteoclasts were reduced (Figure 2B). These results suggested that GKA mainly played an inhibitory role in the early phase of osteoclast differentiation. To explore the effect of GKA on osteoclast functions, the levels of osteoclast marker genes were expressed by RT-PCR (41). The results revealed that the level *DC-STAMP* was severely suppressed after treatment with GKA (Figure 2D), which not only influenced the osteoclast differentiation, but also affected precursor cell fusion. Moreover, the mRNA levels of *Atp6v0d2*, as a typical marker of both precursor cell fusion and bone resorption, was also impaired in a dose-dependent manner (42). Similarly, mRNA levels of TRAP and CTSK were significantly suppressed. All these significant inhibition effect of *c-fos*, *Trap*, *Atp6v0d2*, *ctsk*, and *DC-STAMP* by GKA in a dose-dependent manner indicated that GKA could suppress RANKL-induced osteoclast differentiation.

### Genkwanin Suppressed Bone Resorption *in vitro*

**FIGURE 3:**
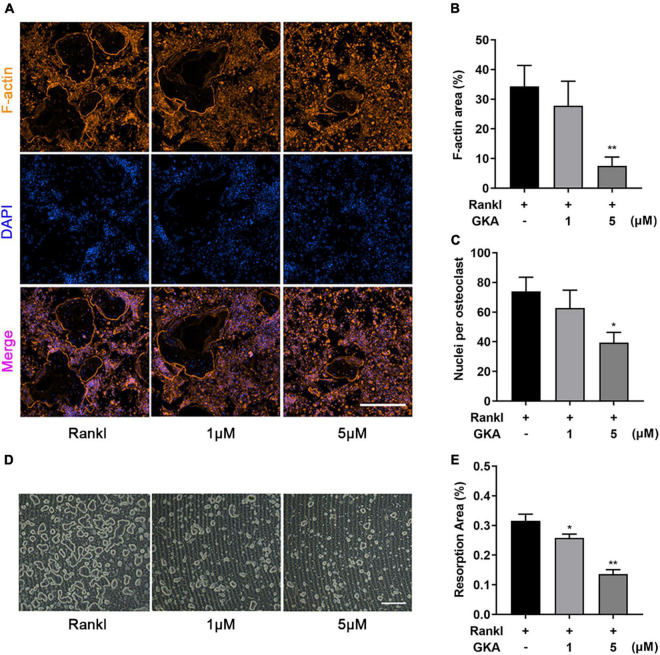
GKA attenuated RANKL-induced osteoclast fusion and bone resorption *in vitro*. **(A)** Representative images of BMMs stained for F-actin and nuclei with Phalloidin-iFluor 488 and DAPI (scale bar, 500 μm). **(B)** Quantification of F-actin area per well. **(C)** The average number of nuclei per osteoclasts. **(D)** Representative images of bone resorption (scale bar, 500 μm). **(E)** The resorption areas were detected using the ImageJ software. The data were illustrated as the Mean ± SD (*P < 0.05, **P < 0.01, compared with the control group; *n* = 3, analyzed by one-way ANOVA).

F-actin ring immunofluorescent staining was used to observe the characteristic cytoskeleton structure of osteoclasts *in vitro*. As shown in Figure 3, GKA inhibited osteoclasts formation and the size of osteoclasts in a dose-dependent manner (Figure 3A). Meanwhile, the F-actin area and average number of nucleus/cell were significantly reduced with the treatment of GKA at the concentration of 5 μM (Figures 3B,C). The resorption assay was performed using bone-mimicking hydroxyapatite-coated substrate plates. Compared with control group, the percentage of bone resorption area in the GKA treatment group was significantly reduced, which indicated that GKA can prevent osteoclast bone resorption (Figures 3D,E). Hence, GKA could attenuate the function of osteoclasts through impairing osteoclast fusion and suppressing bone resorption.

### Genkwanin Suppressed RANKL-Induced p38/c-Fos/NFATc1 and Apoptosis Signaling Pathways *in vitro*

**FIGURE 4:**
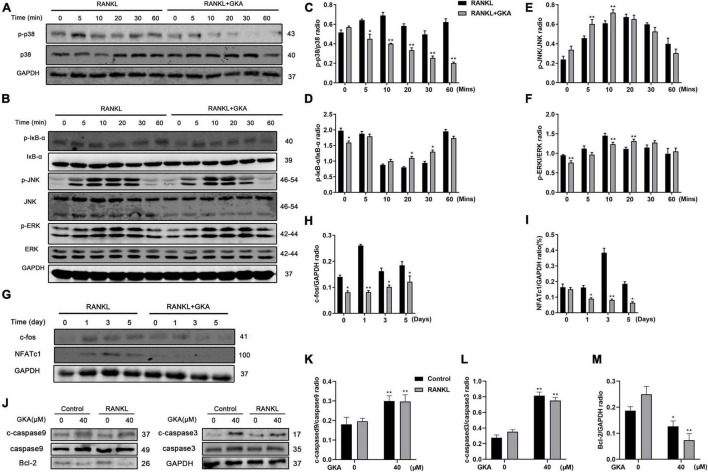
GKA suppressed RANKL-induced p38/c-Fos/NFATc1 and apoptosis signaling pathways. **(A,B)** Representative images of the effects of 5 μM GKA on RANKL-induced p38, IκB-α, JNK, and ERK phosphorylation. **(C–F)** Quantification of the phosphorylated p38, p-IκB-α, p-JNK, p-ERK relative to total p38, IκB-α JNK, and ERK. **(G)** Representative images of effects of 5 μM GKA on RANKL-induced c-fos and NFATc1 for a specific period of time. **(H,I)** Quantification of the radio of NFATc1 and c-Fos relative to GAPDH. **(J)** Representative images of the effects of 40 μM GKA on caspase3, caspase9, and Bcl-2 for 48 h. **(K–M)** Quantification of the radio of cleaved caspase3 and cleaved caspase9 relative to total caspase3 and caspase9 or Bcl-2 relative to GAPDH. The data were presented as the mean ± SD (*P < 0.05, **P < 0.01, analyzed by *t*-test; *n* = 3).

Multiple signaling pathways had been reported to induce RANKL, especially for NF-κB and MAPK signaling pathway (43). Besides, the results in this study suggested that a high dose of GKA induced BMM cell apoptosis, but low dose of GKA inhibited osteoclast differentiation (Figure 1). Hence, western blot was performed to assess the effect of GKA on NF-κB, MAPK, and apoptosis signaling pathways (Figure 4). As shown in Figures 4A,C, the phosphorylation of p38 was significantly attenuated with the treatment of GKA. However, GKA has no significant effect on IκBa, ERK, and JNK (Figure 4B), while phosphorylation of IκBa was activated at 20 and 30 min. In addition, after the treatment of GKA, the phosphorylation of ERK was inhibited at 0 and 10 min (Figures 4D,F), while the phosphorylation of JNK was activated at 5 and 10 min (Figure 4E). Previous studies have reported that c-Fos and NFATc1 were vital transcription factors, which impact osteoclast differentiation and fusion, while p38 regulated the activity of these two transcription factors (44, 45). As expected, these results verified that GKA downregulated the protein levels of c-Fos and NFATc1 (Figures 4G–I). Furthermore, the expression of caspase 3, caspase 9, and Bcl-2 protein in apoptosis signaling pathway was investigated (Figure 4J). Western blot analysis results showed that 40 μM of GKA upregulated cleaved-caspase 3 and cleaved-caspase 9 protein expressions and downregulated Bcl-2 protein level, which indicated that a high dose of GKA induced BMM cell apoptosis (Figures 4K–M). In short, GKA attenuated osteoclast differentiation through inhibiting p38/c-Fos/NFATc1 signaling pathway.

### Genkwanin Did Not Affect Osteoblasts Differentiation *in vitro*

Primary BMSCs were separated from mice tibia and femur, and then, the cell viability was determined. As shown in Supplementary Figure 1A, there was no significant difference between GKA treatment group and control group on cell viability. Subsequently, ALP activity was investigated after the BMSCs were treated with GKA in osteoblast induction medium for 7 days. The results showed that there was no effect on the ALP activity with GKA treatment (Supplementary Figures 1C,D). In addition, RT-PCR results revealed that there is no significance in the expression of osteogenesis genes, including OPG and RANKL after the treatment of GKA *in vivo* (Supplementary Figure 1B). To sum up, GKA had no effect on osteoblasts differentiation.

### Genkwanin Prevented Ovariectomized-Induced Bone Loss *in vivo*

**FIGURE 5:**
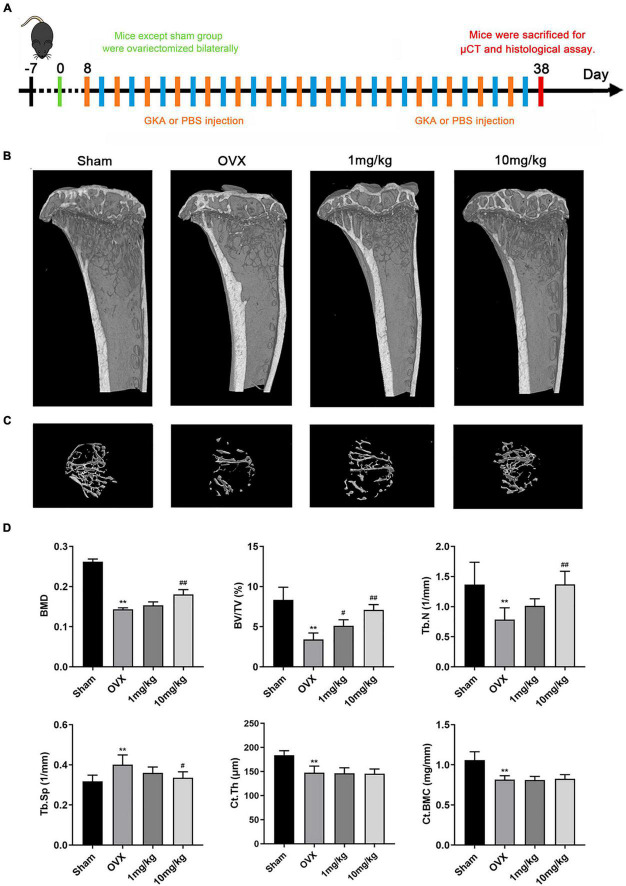
GKA prevented ovariectomy-induced bone loss *in vivo*. **(A)** Treatment regimen. **(B)** Representative images of tibia in sham, OVX, low-dose (1 mg/kg), and high-dose (10 mg/kg) groups. **(C)** 3D reconstructions of the tibial bone in 4 groups. **(D)** Quantification of bone morphometric parameters of BMD, BV/TV, Tb.N, Tb.Sp, Ct.Th, and Ct.BMC in 4 groups. The data were presented as the mean ± SD (**P < 0.01 compared with the sham group, ^#^*P* < 0.05, ^##^*P* < 0.01 relative to the OVX group, analyzed by one-way ANOVA, followed by multiple comparisons per test; *n* = 6).

The bone loss model was performed according to the schedule given in Figure 5A. 3D reconstruction images of tibia in OVX and GKA treatment group showed that GKA protects OVX-induced bone loss (Figures 5B,C). Besides, morphological analysis revealed that BMD, BV/TV, and Tb.N levels were significantly decreased, while Tb.Sp levels were markedly increased after ovariectomy (Figure 5D). Moreover, there was also no significant difference in Ct.Th and Ct.BMC data between the OVX group and the GKA treatment group. The GKA prevented OVX-induced bone loss in mice through inhibiting osteoclastogenesis and bone resorption.

### Genkwanin Inhibited Osteoclastogenesis in Ovariectomized Mice

**FIGURE 6:**
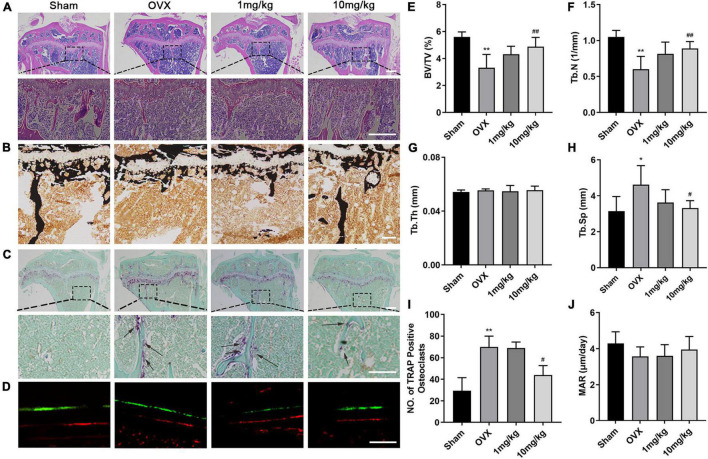
Histological and histomorphometric assessment of the effects of GKA on OVX-induced bone loss. **(A)** Representative histological assessment of tibial bone sections from mice in sham, OVX, low, and high groups stained with hematoxylin and eosin (H&E) (scale bar, 200 μm). **(B)** Microscopic images of longitudinal sections stained with von Kossa (scale bar, 50 μm). **(C)** Representative image of tibial bone from sham, OVX, low, and high groups stained with TRAP (scale bar, 200 μm). **(D)** Representative images of calcein and Alizarin Red (scale bar, 50 μm). **(E–H)** Histomorphometric parameters of BV/TV, Tb.N, Tb.Th, and Tb.Sp. **(I)** Quantitative assessment of the total numbers of TRAP^+^ osteoclasts. **(J)** Histomorphometric parameters of calcein-labeled sections, MAR (mineral apposition rate). The data were presented as the mean ± SD (*P < 0.05, **P < 0.01 relative to the sham group, ^#^*P* < 0.05, ^##^*P* < 0.01 relative to the OVX group, analyzed by one-way ANOVA, followed by multiple comparisons per test; *n* = 6).

We further performed histological analysis, including H&E, Von Kossa, and TRAP staining (Figures 6A–C). Consistent with the abovementioned results, TRAP staining images revealed that GKA reduced the number and activity of TRAP-positive osteoclasts on the bone surface compared with OVX group (Figures 6C,I). Von Kossa staining images suggested that GKA protected the area of the mineralized matrix in a dose-dependent manner. Furthermore, histomorphometric parameters analysis suggested that the value of BV/TV and Tb.N. (mm^–1^) in low and high groups significantly increased, while the value of Tb.Sp decreased. However, Tb.Th. (mm) remained unchanged (Figures 6E–H). In addition, bone dynamics analysis was performed by measuring the level of femoral mineralization matrix formation. The results revealed that MAR was similar among the four groups (Figures 6D,J), which indicated that the inhibition effect of GKA on bone loss was not attributed to the activity of osteoblast, but might be through inhibiting the activity of osteoclasts *in vivo.* Consistent with the results of μCT, the GKA treatment with low and high doses protected mice from bone loss compared with the OVX group. Taken together, GKA was effective on preventing OVX-induced bone loss through inhibiting osteoclastogenesis and bone resorption.

### Genkwanin Protected Lipopolysaccharide-Induced Osteoclast-Mediated Bone Loss *in vivo*

**FIGURE 7:**
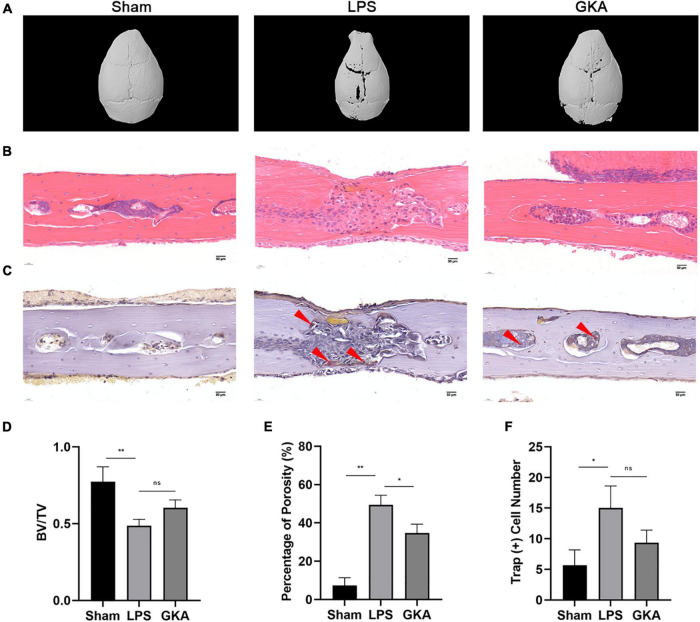
GKA protects against LPS-induced inflammatory osteolysis of mouse calvarium *in vivo.* **(A)** Representative 3D micro-CT reconstructions of mouse calvarium from each group. **(B,C)** Representative hematoxylin-eosin staining (H&E) (100X magnification) and TRAP (100X magnification) stained sections from each group. **(D–F)** Quantitative analysis of **(D)** bone volume to total tissue volume (BV/TV), **(E)** percentage of porosity, and **(F)** the number of TRAP-positive cells were quantified. Values presented as the mean ± standard deviation (*n* = 6); *p < 0.05, **p < 0.01. ns, *P* > 0.05.

The LPS-induced calvarial bone destruction model was used to mimic inflammatory bone destruction. 3D reconstructions of the calvarial bone showed that when compared with the control group, extensive bone destruction, loss of bone volume, and increased bone porosity of the calvarium were observed 7 days after the administration of LPS (Figures 7A–E). Histological assessments further showed abundant inflammatory cell infiltration and elevated numbers of TRAP-positive osteoclasts on the bone surface (Figures 7B,C,F). In contrast, GKA group significantly reduced inflammatory cell infiltration and osteoclast activity (Figure 7).

### Genkwanin Changed the Expression of Cytokines Involved in Osteoclast Formation in Ovariectomized Mice

We examined inflammation cytokines in BMM cells induced by IL-1β and treated with GKA (1 μM and 5 μM), as shown in Figure 1D, GKA suppressed the expression of TNF-α and IL-6 in a concentration dependent manner. Previous studies have been reported that GKA reduced pro-inflammatory mediators and the expression of cytokines enhanced osteoclast formation in the OVX model (31). The results showed that the levels of IL-6, TNF-α, and IL-1β were increased after OVX construction, whereas these inflammatory factors were decreased with the treatment of GKA, which will inhibit the osteoclast formation (Supplementary Figure 1E).

## Discussion and Conclusion

Bone tissues in the human body keep a dynamic balance along with the bone resorption and bone formation. Osteoblasts are vital for bone formation, while osteoclasts are significant for bone resorption. When osteoclasts are overproduced, excessive bone resorption occurred and resulted in osteoporosis (46). Moreover, the decrease of the estrogen levels after menopause could overactivate osteoclasts formation, thereby exacerbating bone resorption. Therefore, the decrease of estrogen levels is a main factor leading to postmenopausal osteoporosis (7, 47–49). The current drugs used in clinical, for example, bisphosphonates and selective estrogen receptor modulators, have positive effects on osteoporosis, but they still have some side effects (11, 50). Thus, it is still a big challenge to explore new drugs for postmenopausal osteoporosis.

Genkwanin, isolated from *G. Flos, Psychotria serpens L*, and the leaves of *Cistus laurifolius L*, has been widely applied as an anti-inflammatory and immunomodulatory agent. However, less research has been done to probe the effect of GKA on osteoclastogenesis and bone resorption. Hence, we investigated whether GKA influence the osteoclastogenesis *in vitro* and OVX-induced bone loss *in vivo*. The results revealed that GKA suppressed the differentiation of osteoclasts induced by RANKL *in vitro.* Moreover, bone loss was dramatically reduced by GKA treatment in OVX mouse model *in vivo*.

Osteoclasts, a kind of multinucleated cells, were formed on the basis of the action of two key cytokines, including M-CSF and RANKL (6), in which M-CSF is mainly responsible for the viability of osteoclast precursors, whereas RANKL activates a series of signaling pathways after combining with RANK. Therefore, osteoclast precursors differentiate into mature osteoclasts for bone resorption. Briefly, when RANKL binds to RANK, the MAPK signaling pathway was activated. The MAPK superfamily members consist of p38, JNK, and ERK (51). When the p38 signaling pathway is activated, c-Fos, as a main component of AP-1 downstream, is also activated (52, 53). Furthermore, c-Fos combines with the promoter region of NFATc1, the main regulator of osteoclast formation, thereby regulating NFATc1 to activate osteoclast differentiation, survival, and bone resorption (54, 55). In turn, when the p38 signaling pathway is inhibited, the formation of osteoclasts will be inhibited, thereby inhibiting bone resorption caused by excessive activation of osteoclast (56–58). Previous studies have shown that certain compounds could bind to the ATP-binding pocket of p38, further inhibiting the phosphorylation of p38 (59, 60). Therefore, it might be a kind of hypothesis that GKA interacts with the ATP-binding pocket of p38, thereby inhibiting the phosphorylation of p38. In addition, researchers reported that inhibitors could also indirectly affect the conformation of ATP sites by binding to adjacent positions, further inhibiting p38 phosphorylation. Therefore, it is also another kind of hypothesis that GKA affects the phosphorylation of p38 by binding to the position adjacent to the ATP site (61, 62). In this research, we demonstrated that low-dose GKA suppressed osteoclast differentiation by inhibiting the phosphorylation of p38, thereby inhibiting c-Fos and NFATc1, whereas high-dose GKA induced BMMs apoptosis. Meanwhile, the phosphorylation level of ERK was also decreased with the treatment of GKA. RT-PCR results also revealed that GKA inhibited the mRNA levels of *Trap*, *c-fos*, *ctsk, DC-STAMP*, and *Atp6v0d2*. In summary, the results revealed that GKA could inhibit the osteoclastogenesis through inhibiting the p38 and ERK/MAPK signaling pathway in the early differentiation stage, which might be used for the treatment of postmenopausal osteoporosis. *In vitro* experiments demonstrated the inhibition effect of GKA on osteoclast. Hence, it was further studied whether GKA has a therapeutic effect on the osteolytic bone loss induced by LPS and OVX *in vivo*. Consistent with the results of *in vitro* experiments, the bone loss indexes, including BV/TV, Tb.Th, Tb.N, and Conn.D, were significantly improved after GKA treatment. Therefore, it could be concluded that GKA reduced the total amount of osteoclasts and density of osteoclasts per bone surface, which has the potential to treat osteoporosis in large animal models, even in humans. In addition, the osteoclasts are used for promoting bone resorption, while osteoblasts are used for improving bone formation, which maintains the balance of bone homeostasis (63, 64). The results suggested that GKA mainly inhibited the osteoclastogenesis and the bone resorption activities and hardly affected the formation of osteoblasts to reverse bone loss.

In conclusion, the therapeutic effect of GKA on osteoclasts was mainly achieved through inhibiting the differentiation of osteoclasts induced by RANKL. Moreover, the underlying mechanism might be that the osteoclast precursors were differentiated into mature osteoclasts for bone resorption through the inhibition of the MAPK signaling pathway activation. In addition, results of *in vivo* experiments revealed that GKA could prevent OVX and LPS-induced bone loss, which provided some promising evidence for the use of GKA in the prevention or therapeutic treatment of osteoclast-mediated osteolytic bone diseases.

## Data Availability Statement

The original contributions presented in this study are included in the article/Supplementary Material, further inquiries can be directed to the corresponding authors.

## Ethics Statement

The animal study was reviewed and approved by Ethics Committee of Shanghai University.

## Author Contributions

XF, XS, and CZ mainly performed the design and conception of this study. XF and JW performed experiments. XS, CZ, NL, HG, CX, and JL were involved *in vitro* and *in vivo* experiments. LS, ML, and XZ conceived to the study. XF wrote the manuscript. All authors discussed the results, commented on the manuscript, and contributed to the writing and submission of the manuscript.

## Conflict of Interest

The authors declare that the research was conducted in the absence of any commercial or financial relationships that could be construed as a potential conflict of interest.

## Publisher’s Note

All claims expressed in this article are solely those of the authors and do not necessarily represent those of their affiliated organizations, or those of the publisher, the editors and the reviewers. Any product that may be evaluated in this article, or claim that may be made by its manufacturer, is not guaranteed or endorsed by the publisher.

## Supplementary Material

Supplementary Figure 1 GKA has no inhibitory effect on osteoclast differentiation. **(A)** Viability of BMSCs was assessed by CCK-8 assay after treating with GKA for 48 h. **(B)** The mRNA expression of OPG and RANKL in bone was quantified. **(C)** The effect of GKA on osteoblast differentiation. BMSCs were cultured in osteogenic induction media with or without specified concentrations of anacardic acid for 7 days before cell fixation and staining to detect ALP activity (scale bar, 200 μm). **(D)** ALP activity relative to untreated controls was calculated. **(E)** The levels of inflammatory cytokines in the serum. The data were demonstrated as the mean ± SD (^∗^*P* < 0.05, ^∗∗^*P* < 0.01, relative to the control group; *n* = 3 per group, analyzed by one-way ANOVA).
