# Supplementary figures and images for: Genkwanin Prevents Lipopolysaccharide-Induced Inflammatory Bone Destruction and Ovariectomy-Induced Bone Loss
Source: Front Nutr. 2022 Jun 23;9:921037. doi: 10.3389/fnut.2022.921037 (PMC9260391; doi:10.3389/fnut.2022.921037)

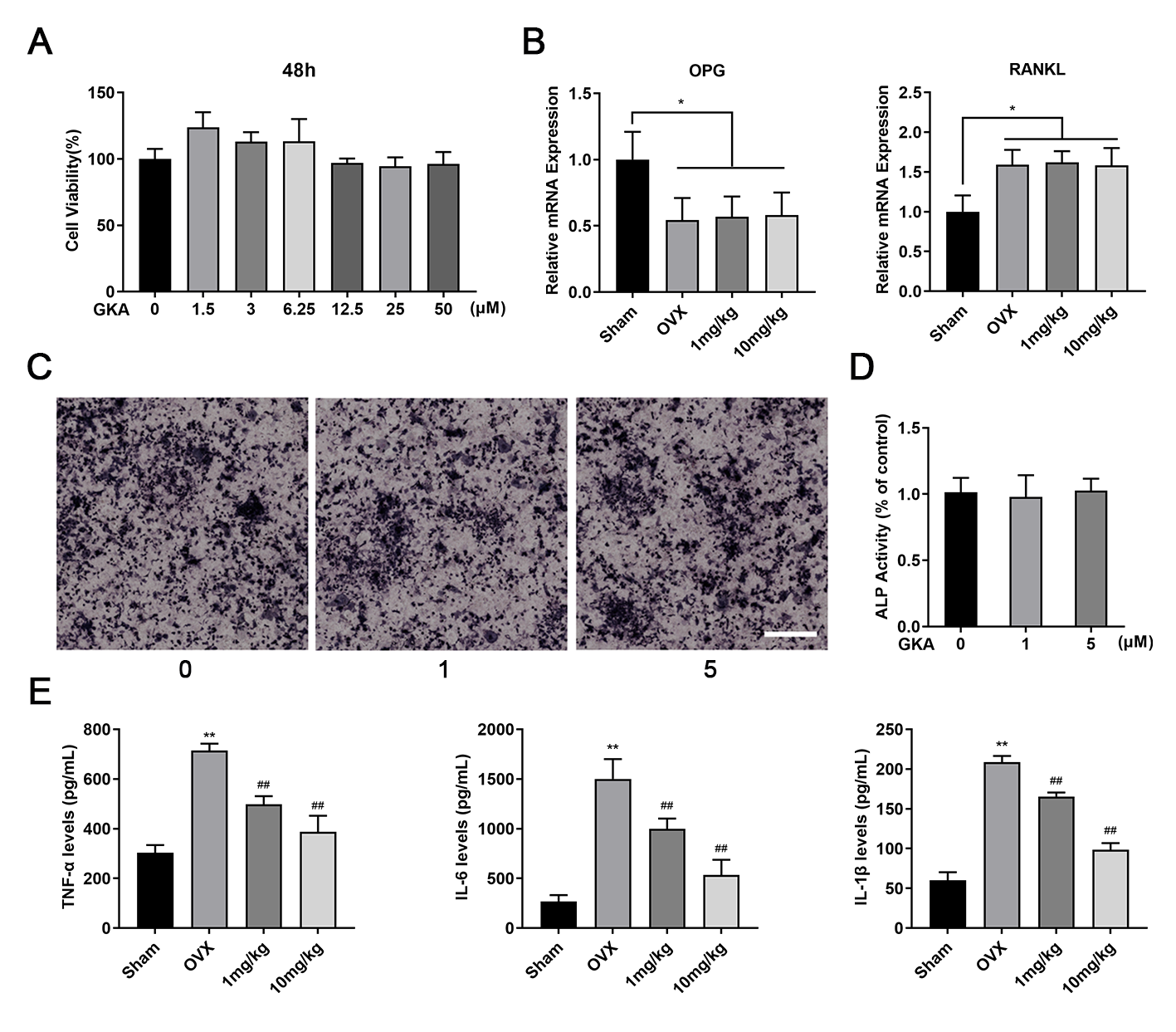

Supplement: Supplementary Figure 1 — GKA has no inhibitory effect on osteoclast differentiation. (A) Viability of BMSCs was assessed by CCK-8 assay after treating with GKA for 48 h. (B) The mRNA expression of OPG and RANKL in bone was quantified. (C) The effect of GKA on osteoblast differentiation. BMSCs were cultured in osteogenic induction media with or without specified concentrations of anacardic acid for 7 days before cell fixation and staining to detect ALP activity (scale bar, 200 μm). (D) ALP activity relative to untreated controls was calculated. (E) The levels of inflammatory cytokines in the serum. The data were demonstrated as the mean ± SD (∗P < 0.05, ∗∗P < 0.01, relative to the control group; n = 3 per group, analyzed by one-way ANOVA). [file Image_1.TIF]

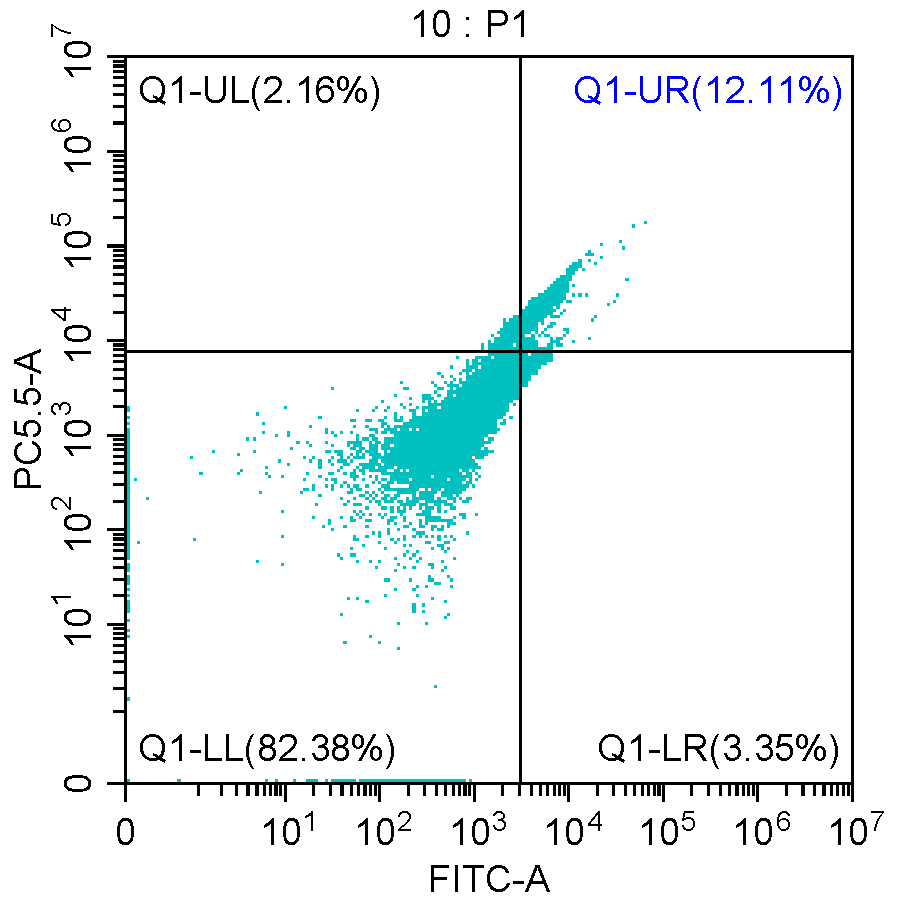

Supplement: Supplementary file 9 [file Data_Sheet_1.ZIP › apoptosis/10_Plot1.bmp]

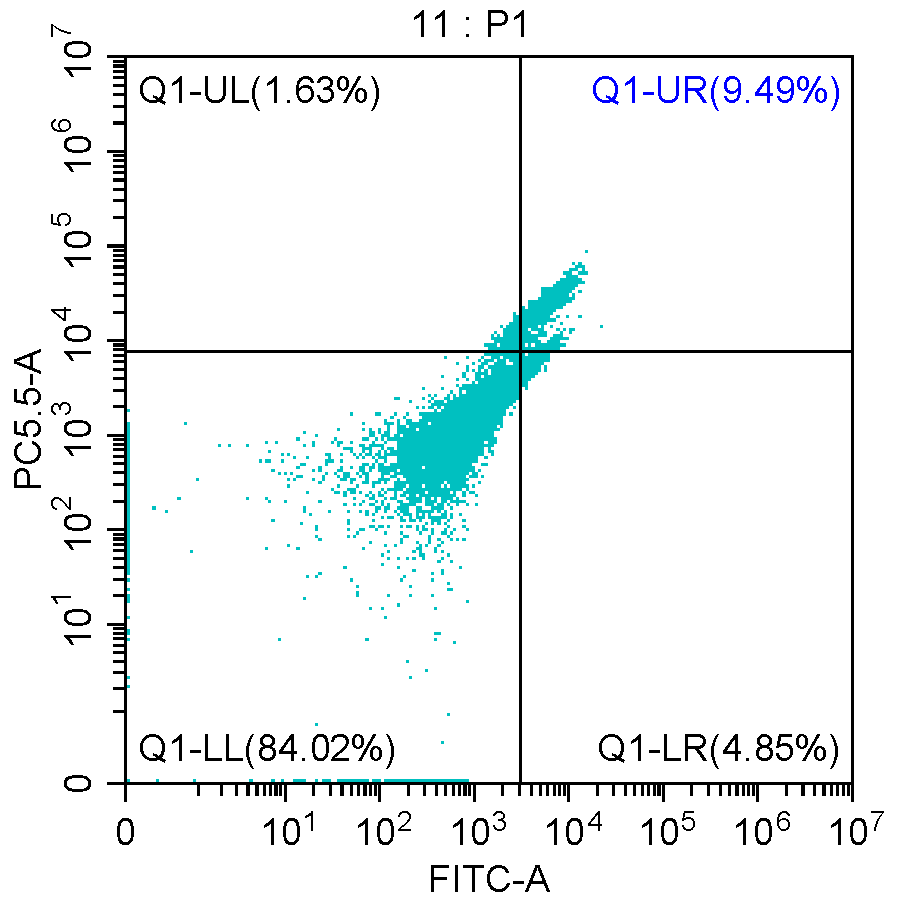

Supplement: Supplementary file 9 [file Data_Sheet_1.ZIP › apoptosis/11_Plot1.bmp]

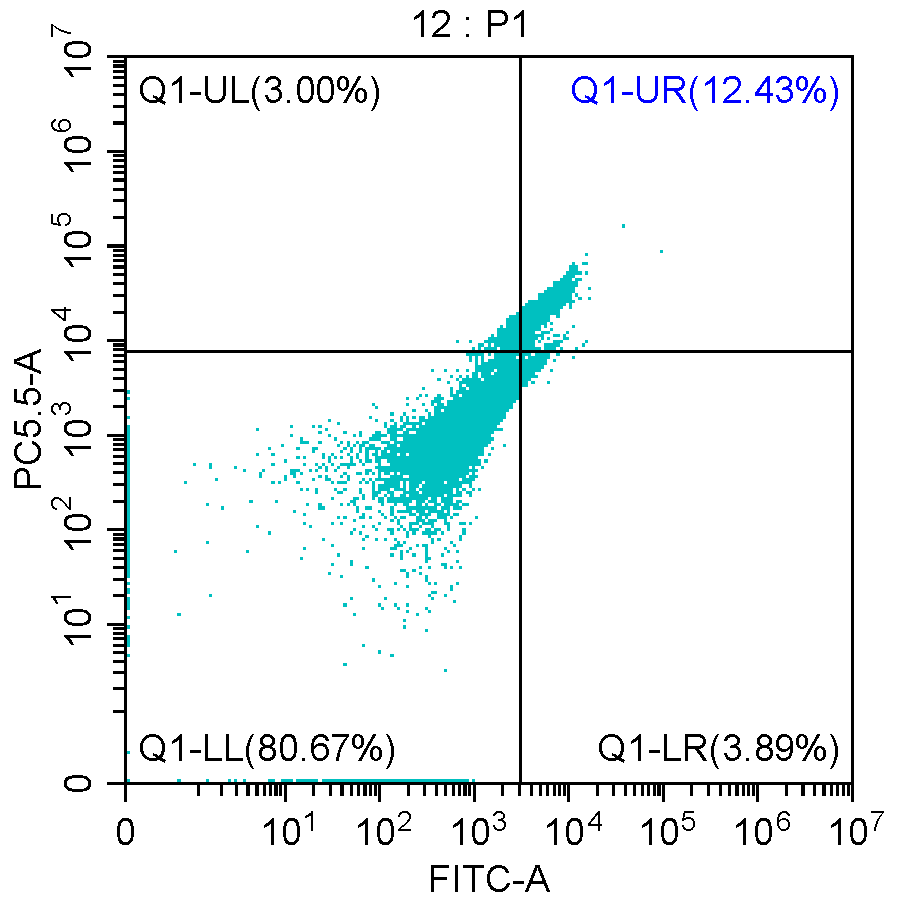

Supplement: Supplementary file 9 [file Data_Sheet_1.ZIP › apoptosis/12_Plot1.bmp]

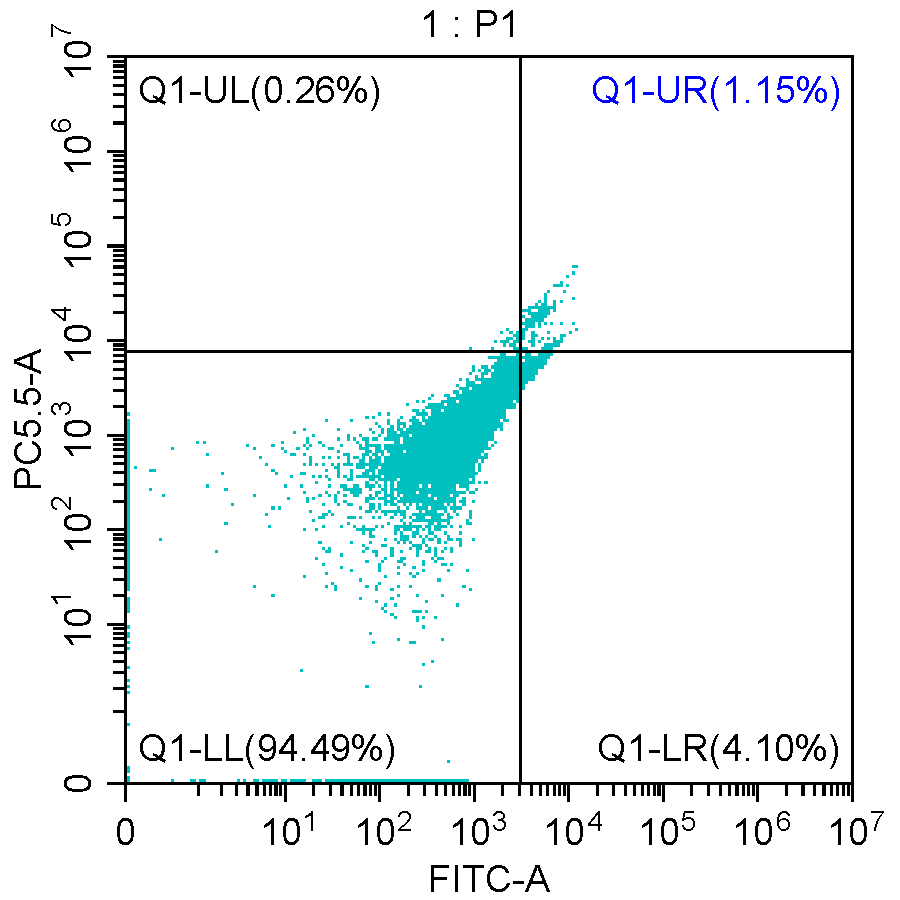

Supplement: Supplementary file 9 [file Data_Sheet_1.ZIP › apoptosis/1_Plot1.bmp]

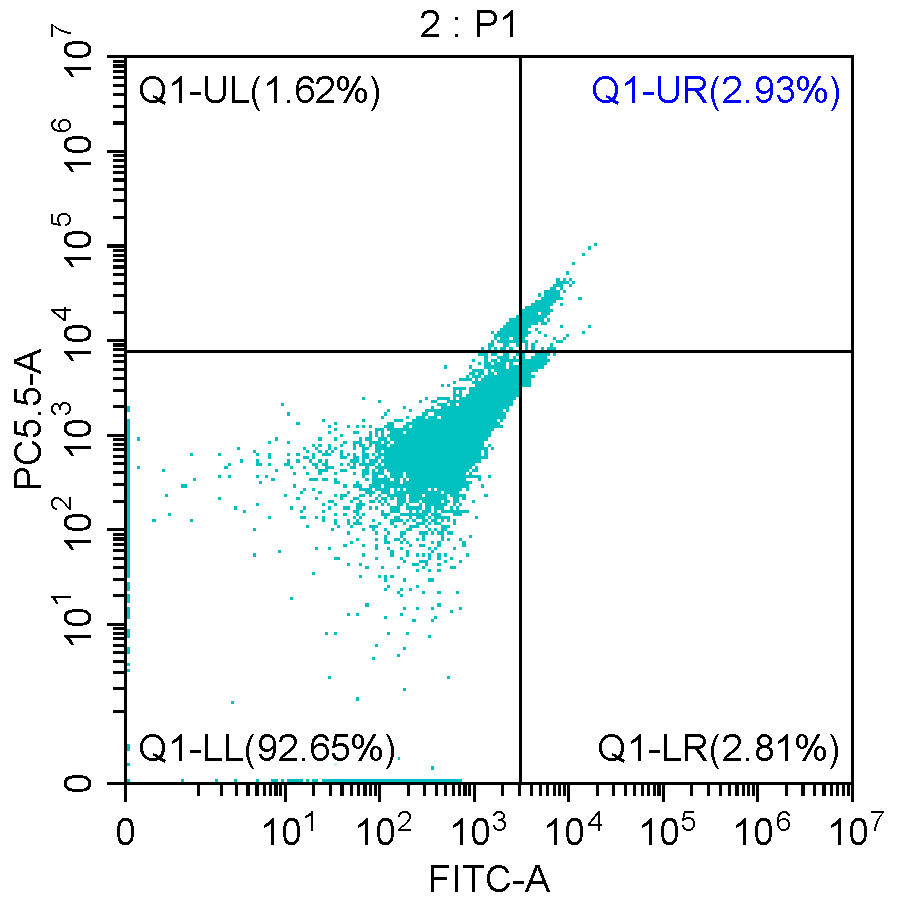

Supplement: Supplementary file 9 [file Data_Sheet_1.ZIP › apoptosis/2_Plot1.bmp]

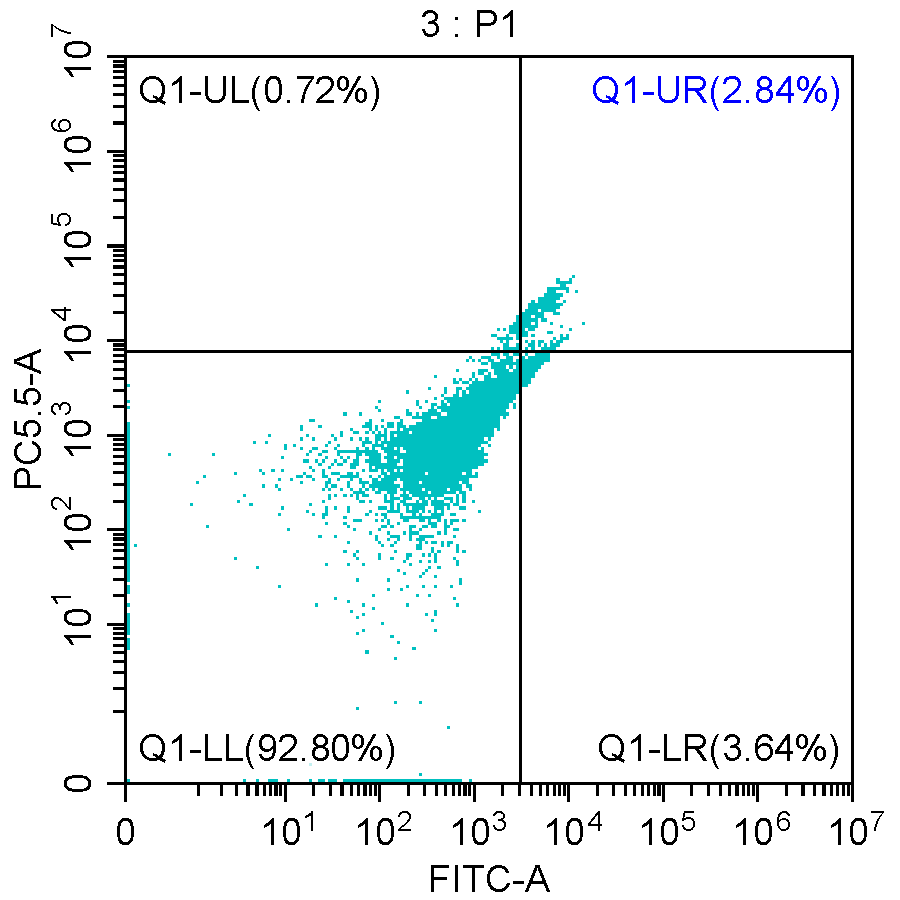

Supplement: Supplementary file 9 [file Data_Sheet_1.ZIP › apoptosis/3_Plot1.bmp]

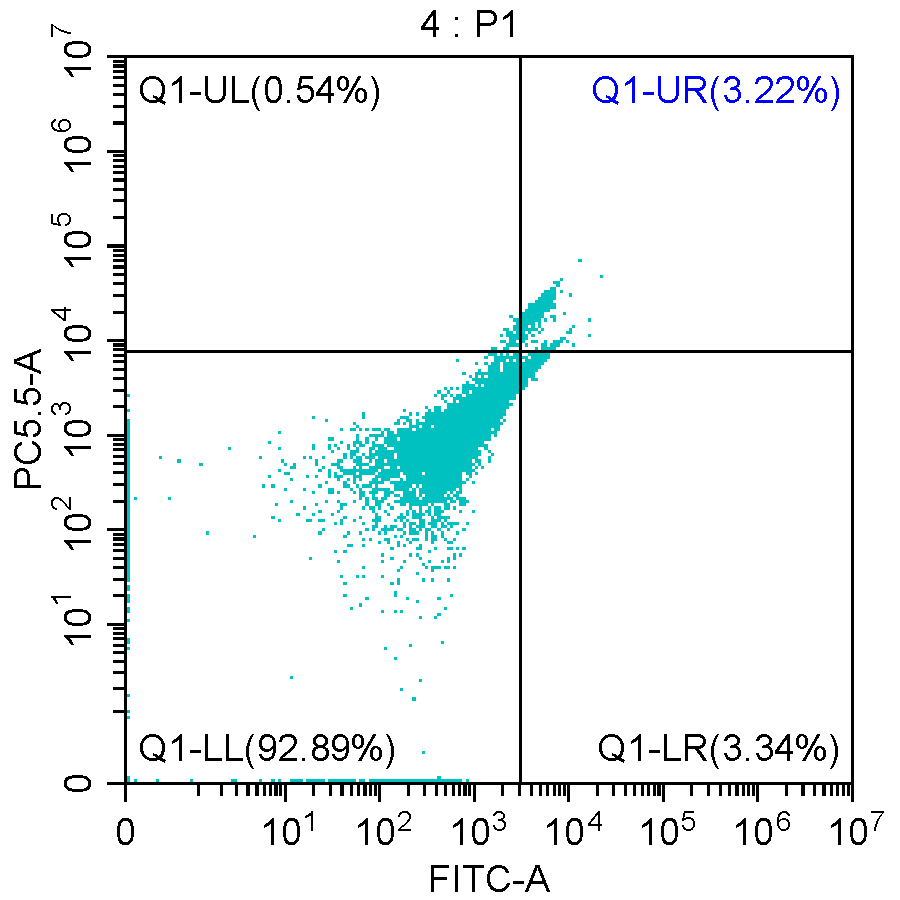

Supplement: Supplementary file 9 [file Data_Sheet_1.ZIP › apoptosis/4_Plot1.bmp]

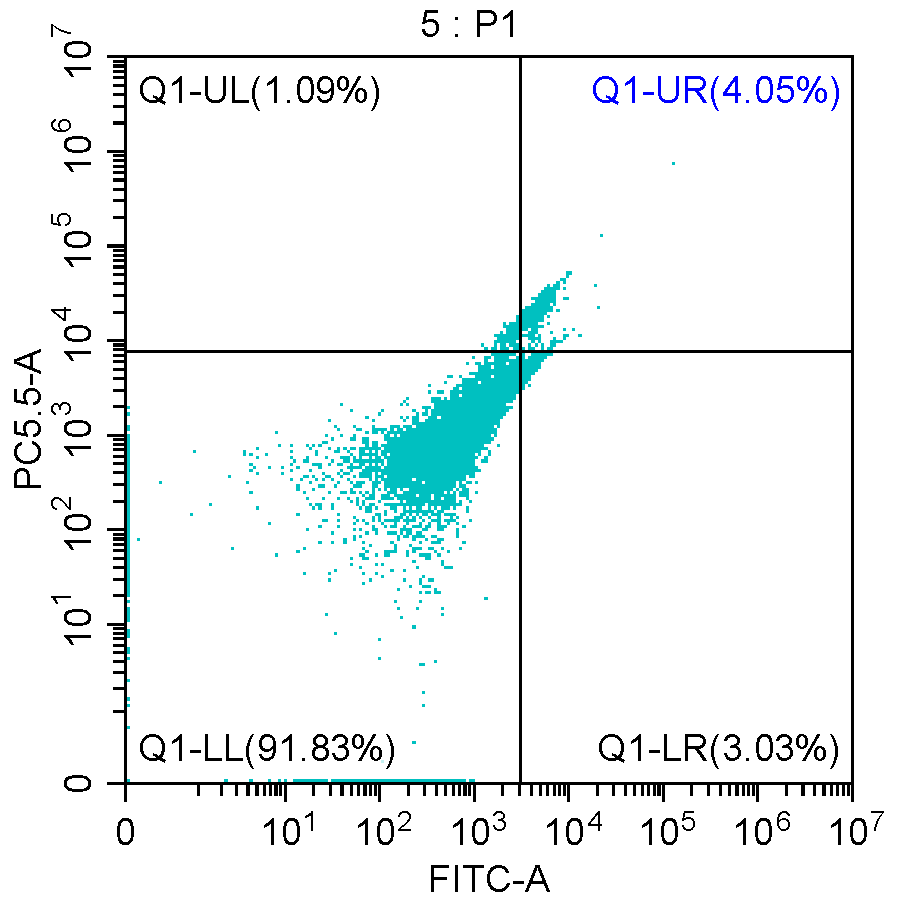

Supplement: Supplementary file 9 [file Data_Sheet_1.ZIP › apoptosis/5_Plot1.bmp]

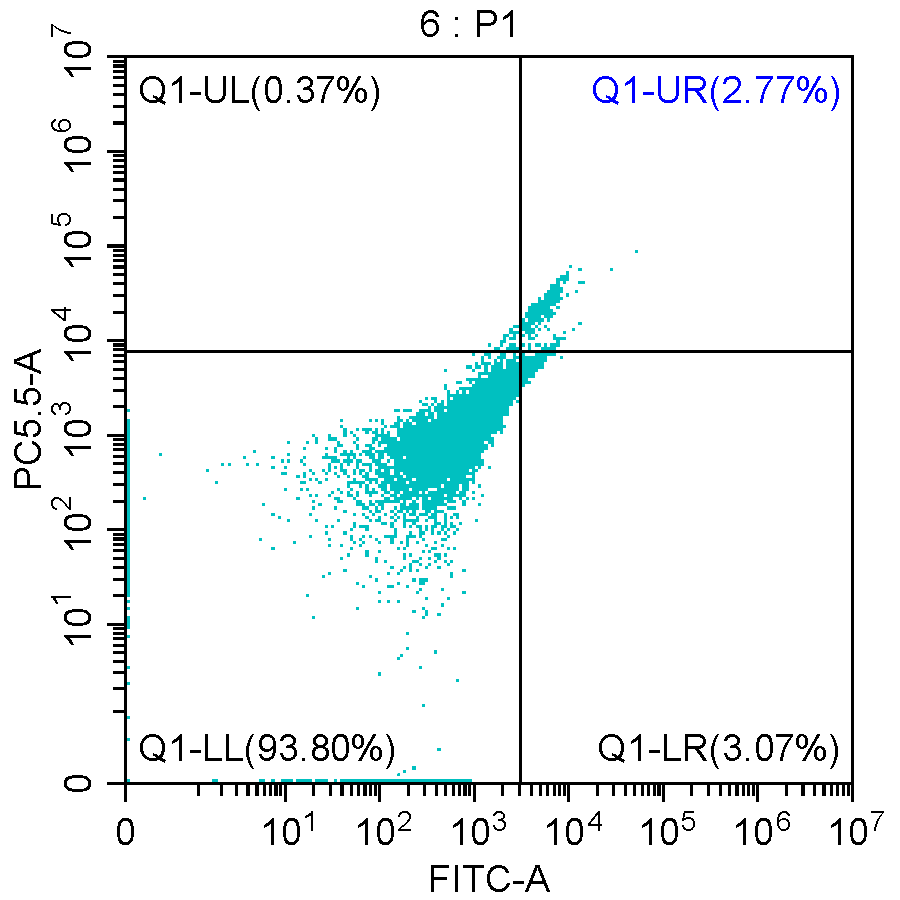

Supplement: Supplementary file 9 [file Data_Sheet_1.ZIP › apoptosis/6_Plot1.bmp]

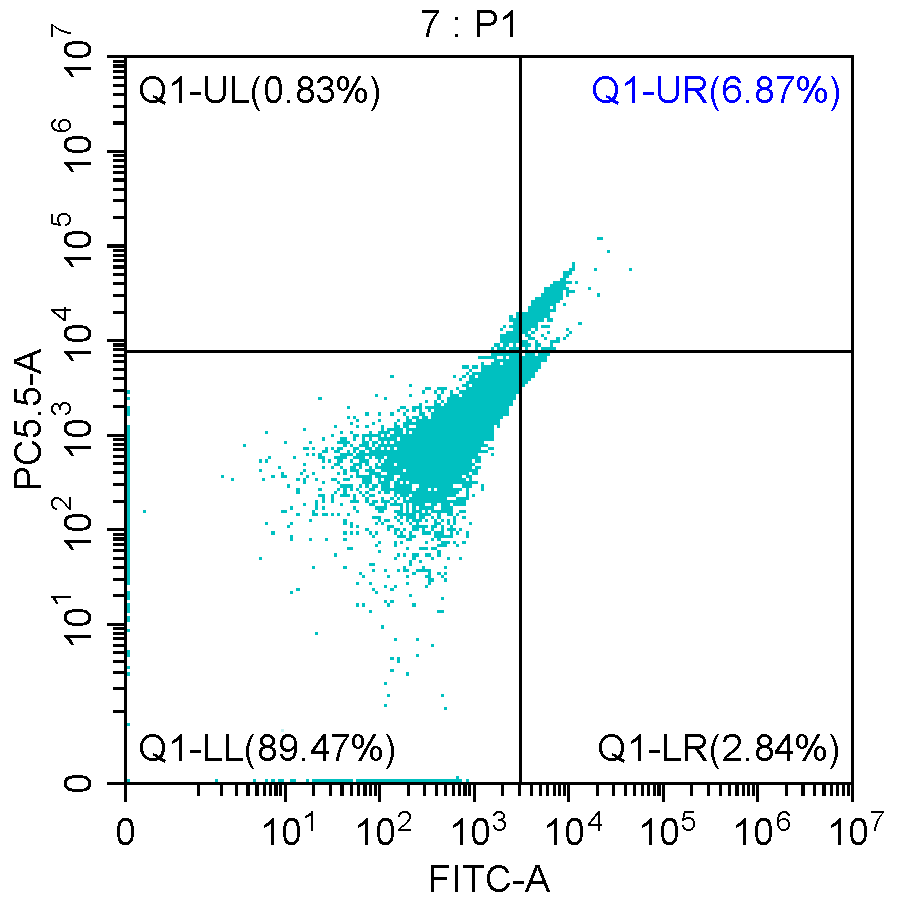

Supplement: Supplementary file 9 [file Data_Sheet_1.ZIP › apoptosis/7_Plot1.bmp]

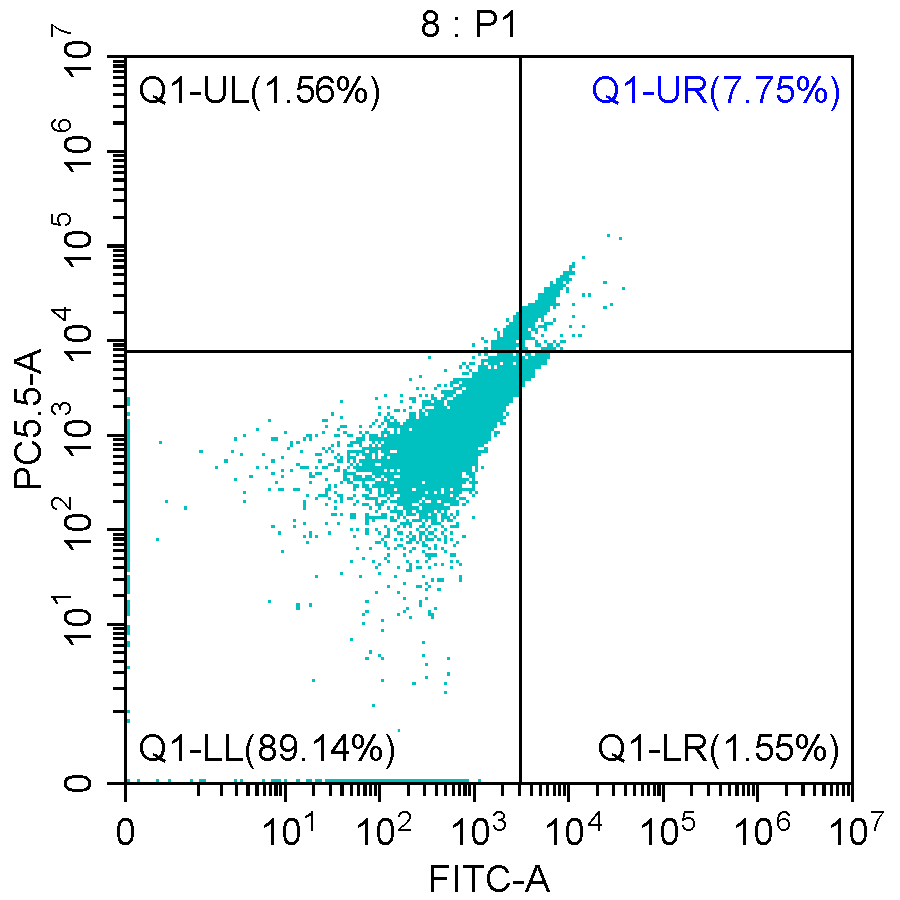

Supplement: Supplementary file 9 [file Data_Sheet_1.ZIP › apoptosis/8_Plot1.bmp]

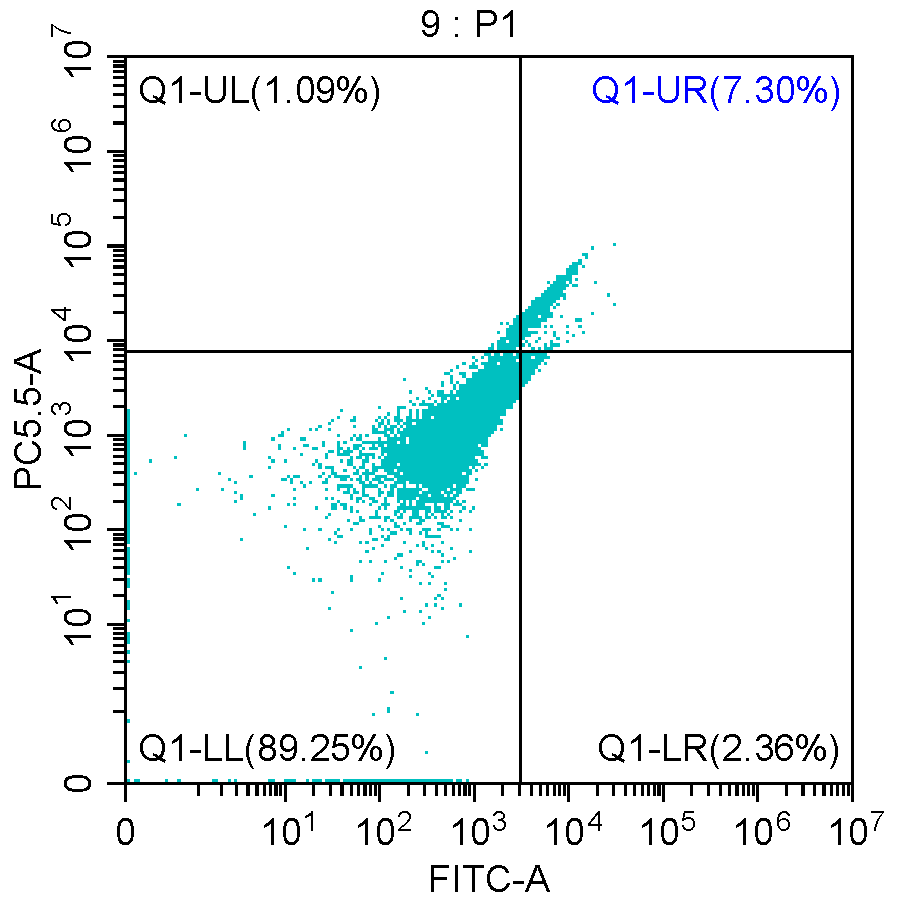

Supplement: Supplementary file 9 [file Data_Sheet_1.ZIP › apoptosis/9_Plot1.bmp]

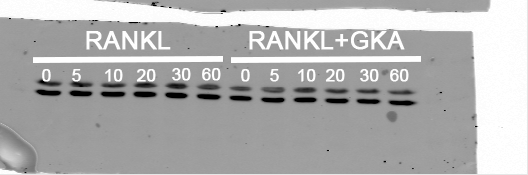

Supplement: Supplementary file 10 [file Data_Sheet_2.ZIP › fig4/ERK.tif]

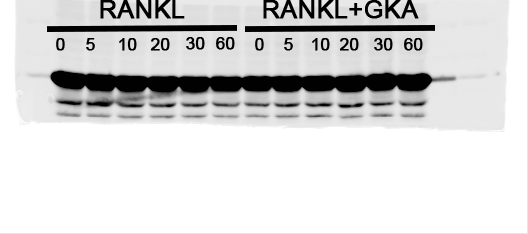

Supplement: Supplementary file 10 [file Data_Sheet_2.ZIP › fig4/GAPDH (1).tif]

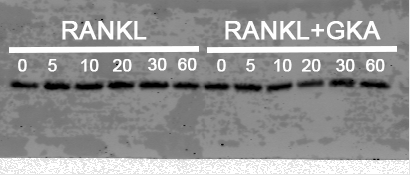

Supplement: Supplementary file 10 [file Data_Sheet_2.ZIP › fig4/GAPDH (2).tif]

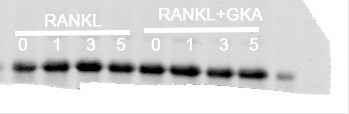

Supplement: Supplementary file 10 [file Data_Sheet_2.ZIP › fig4/GAPDH.tif]

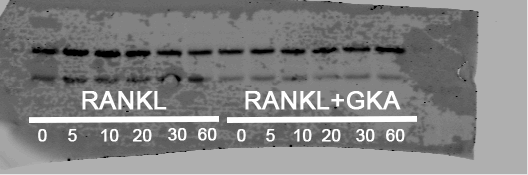

Supplement: Supplementary file 10 [file Data_Sheet_2.ZIP › fig4/JNK.tif]

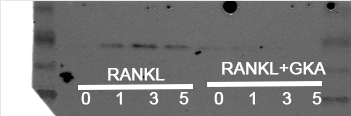

Supplement: Supplementary file 10 [file Data_Sheet_2.ZIP › fig4/NFATc1.tif]

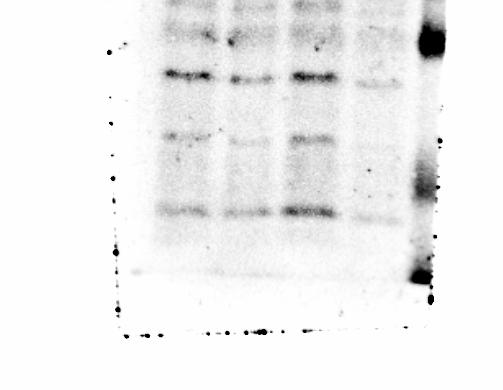

Supplement: Supplementary file 10 [file Data_Sheet_2.ZIP › fig4/bcl-2(IRDye 800CW).tif]

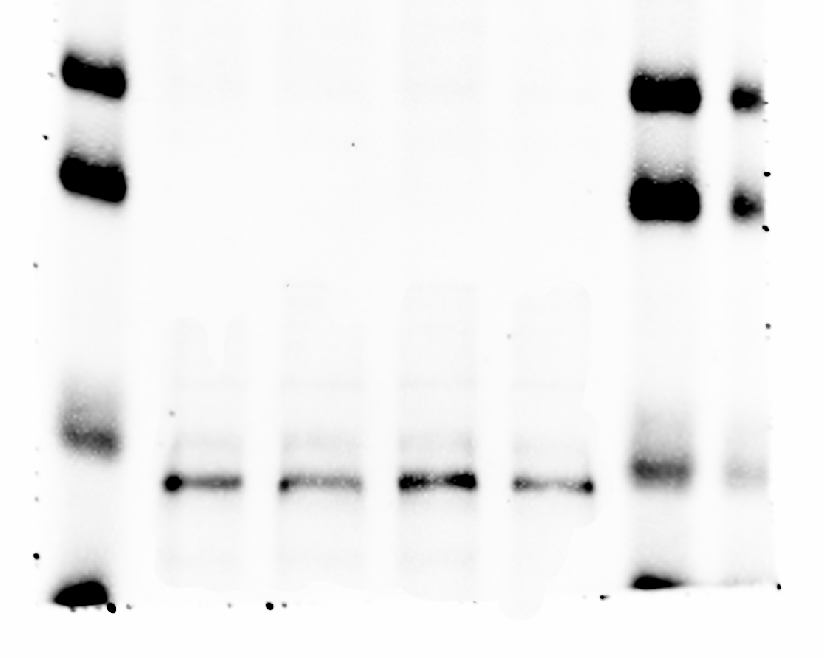

Supplement: Supplementary file 10 [file Data_Sheet_2.ZIP › fig4/c-caspase3(IRDye 800CW).tif]

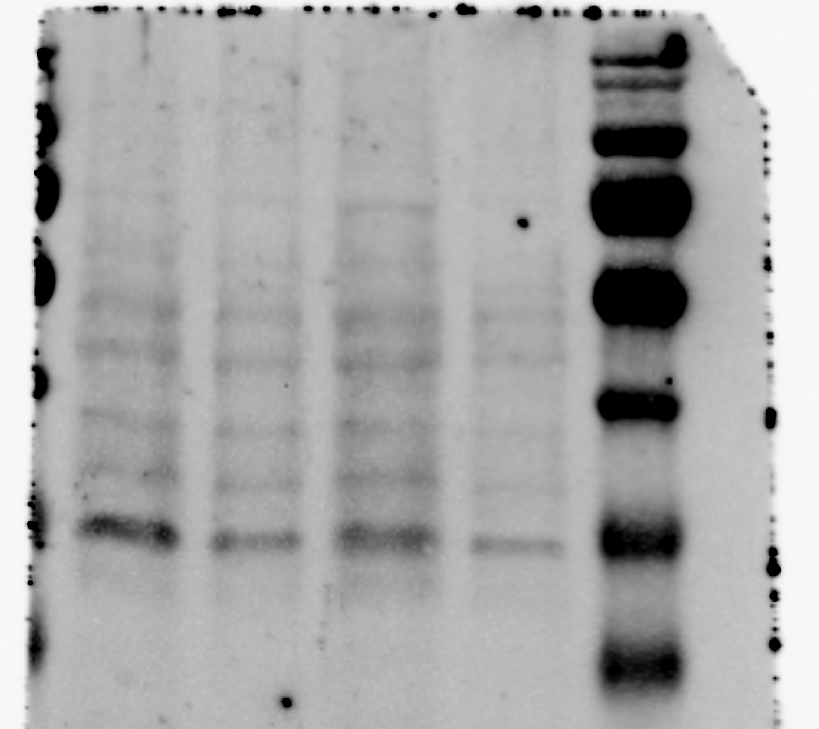

Supplement: Supplementary file 10 [file Data_Sheet_2.ZIP › fig4/c-caspase9(IRDye 800CW).tif]

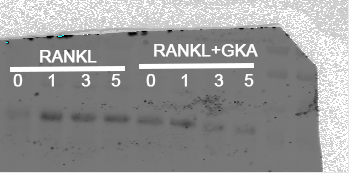

Supplement: Supplementary file 10 [file Data_Sheet_2.ZIP › fig4/c-fos.tif]

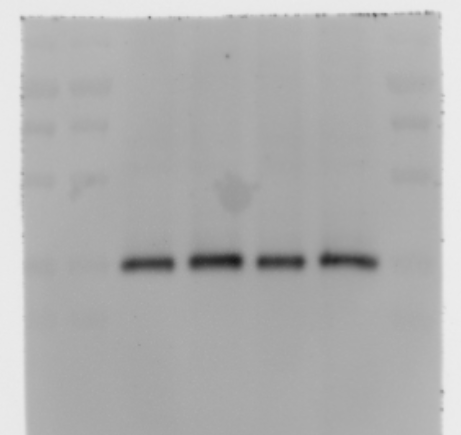

Supplement: Supplementary file 10 [file Data_Sheet_2.ZIP › fig4/caspase-3(IRDye 800CW).tif]

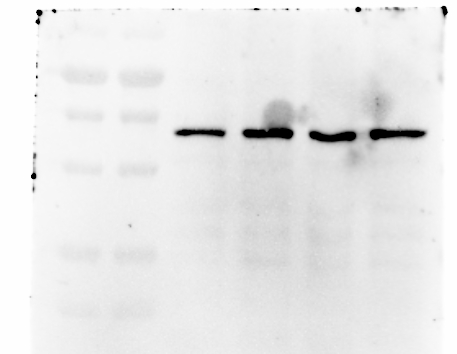

Supplement: Supplementary file 10 [file Data_Sheet_2.ZIP › fig4/caspase-9(IRDye 800CW).tif]

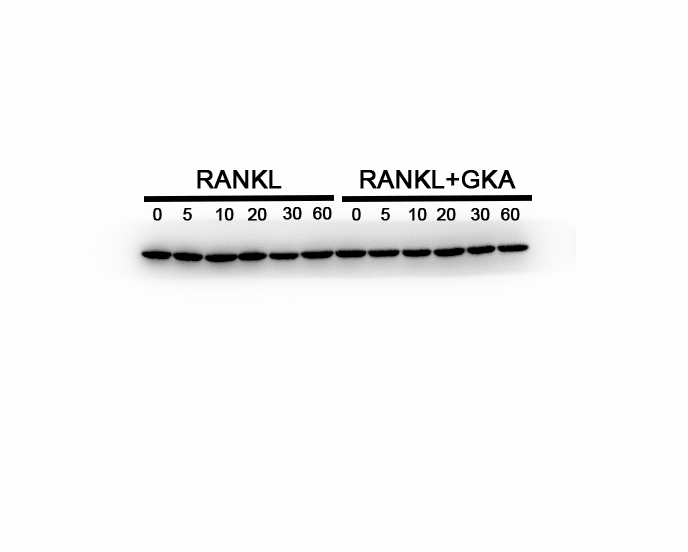

Supplement: Supplementary file 10 [file Data_Sheet_2.ZIP › fig4/la╩B-a┴.tif]

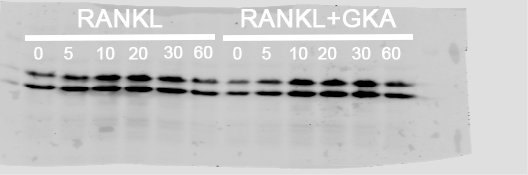

Supplement: Supplementary file 10 [file Data_Sheet_2.ZIP › fig4/p-ERK.tif]

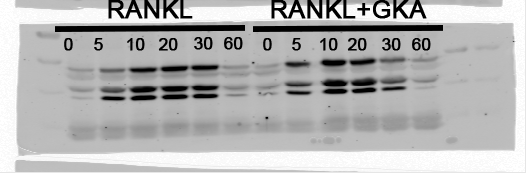

Supplement: Supplementary file 10 [file Data_Sheet_2.ZIP › fig4/p-JNK.tif]

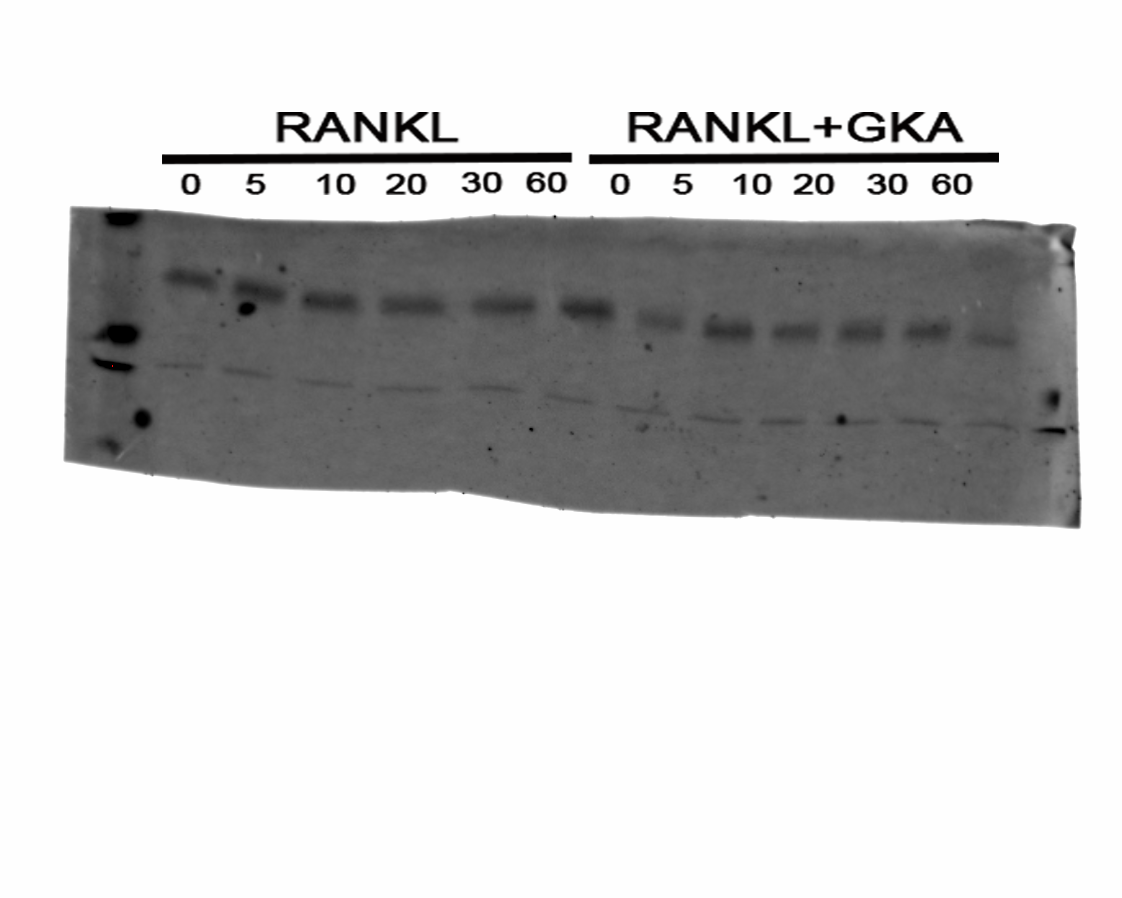

Supplement: Supplementary file 10 [file Data_Sheet_2.ZIP › fig4/p-la╩B-a┴.tif]

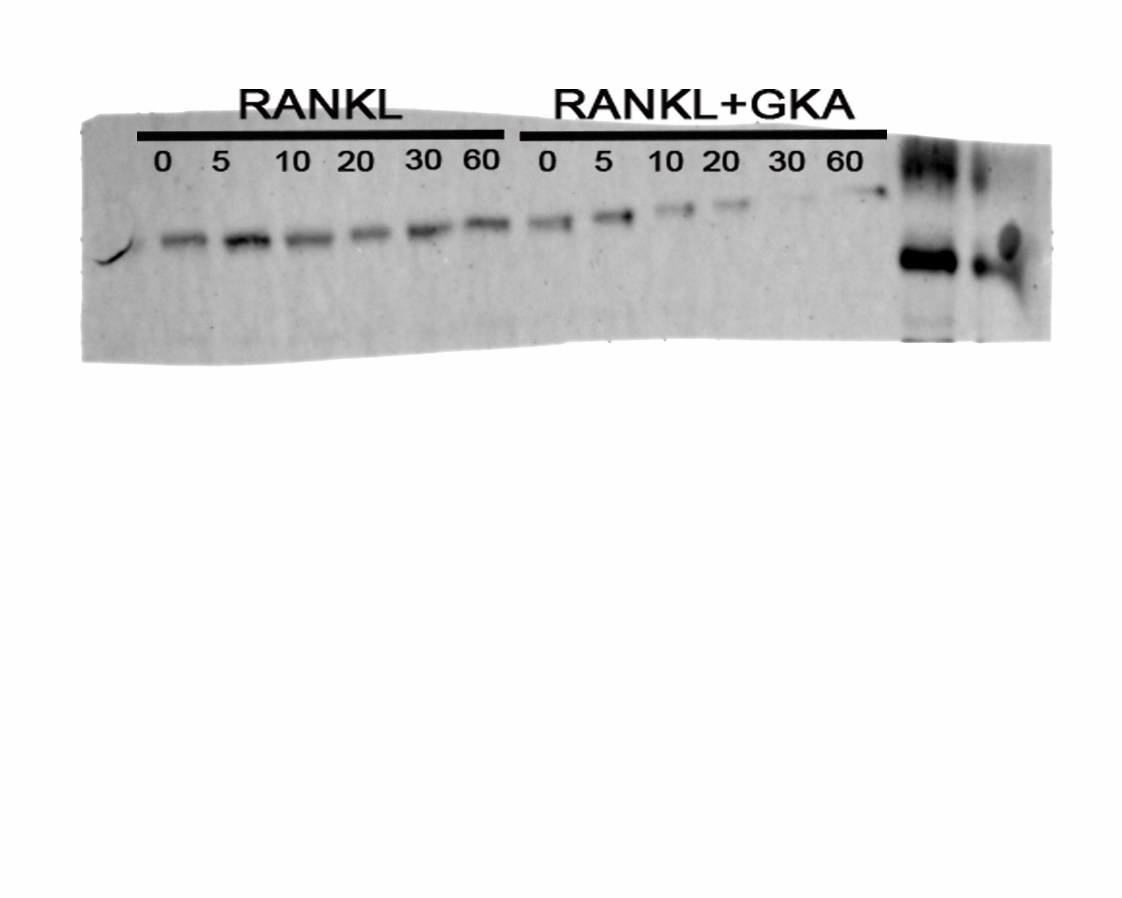

Supplement: Supplementary file 10 [file Data_Sheet_2.ZIP › fig4/p-p38.tif]

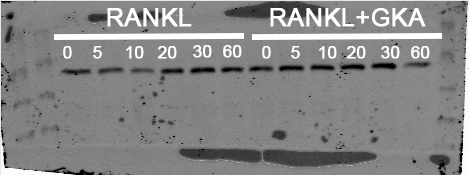

Supplement: Supplementary file 10 [file Data_Sheet_2.ZIP › fig4/p38.tif]
